# The Machine Learning Classification of Retinal Ganglion Cell Dendritic Texture in a 3xTg-Alzheimer’s Disease Mouse Model

**DOI:** 10.3390/diagnostics16162672

**Published:** 2026-08-21

**Authors:** Mukhit Kulmaganbetov, Saken Khaidarov, Ryan Bevan, James E. Morgan

**Affiliations:** 1 School of Optometry and Vision Sciences, Cardiff University, Cardiff CF24 4HQ, UK; bevanrj@cardiff.ac.uk (R.B.); morganje3@cardiff.ac.uk (J.E.M.); 2 Department of Molecular Biology and Medical Genetics, Asfendiyarov Kazakh National Medical University, Almaty 050000, Kazakhstan; khaidarov.s@kaznmu.kz; 3 Centre for Eye and Vision Research, Hong Kong SAR, China; 4 Centre for Sanitary and Epidemiological Expertise, Medical Centre of the Presidential Administration of the Republic of Kazakhstan, Astana 010000, Kazakhstan; 5 UK Dementia Research Institute at Cardiff, Cardiff University, Cardiff CF24 4HQ, UK; 6 Systems Immunity Research Institute, Cardiff University, Cardiff CF24 4HQ, UK; 7 University Hospital of Wales, Heath Park, Cardiff CF14 4XW, UK

**Keywords:** optical coherence tomography, texture analysis, grey-level co-occurrence matrix, support vector machine, retinal ganglion cell, inner plexiform layer, Alzheimer’s disease, 3xTg-AD, retinal biomarker, machine learning

## Abstract

**Background/Objectives:** Retinal imaging has considerable potential for monitoring Alzheimer’s disease (AD) neurodegeneration, as retinal ganglion cell dendritic atrophy within the inner plexiform layer (IPL) is an early event. We tested whether quantitative optical coherence tomography (OCT) speckle texture analysis combined with supervised machine learning could discriminate AD-related IPL alterations without exogenous contrast agents in a mouse model. **Methods:** Retinal explants from triple-transgenic AD mice (*n* = 7, aged 12 months) and C57BL/6 controls (*n* = 3, aged 15 months) were imaged ex vivo using a custom 1040 nm spectral-domain OCT system. Five grey-level co-occurrence matrix (GLCM) features were extracted from IPL volumes of interest (VOIs) and classified using a linear support vector machine (SVM). **Results:** AD and control IPL textures formed two completely separable clusters in a two-dimensional feature space defined by contrast and entropy (0°), achieving 100% VOI-level classification accuracy (95% CI: 96.4–100%). However, given the small sample size, VOI-level rather than animal-level validation, lack of histological confirmation, non-interleaved image acquisition, and differences in age/strain between groups, these results represent exploratory dataset separability rather than a validated diagnostic test. **Conclusions:** These findings demonstrate the feasibility of the ligand-free, texture-based OCT discrimination of IPL alterations, indicating a strong underlying optical signal. Adequately powered, in vivo longitudinal studies with matched controls, interleaved acquisition, animal-level cross-validation, and histological validation are required before any clinical translation.

## 1. Introduction

Alzheimer’s disease (AD) remains the leading cause of dementia [1,2], and its defining pathologies, such as the accumulation of β-amyloid [3] and microtubule-associated tau protein [4] and progressive synaptic and neuronal loss [5], typically precede clinical symptoms by several years [6]. Methods for monitoring changes during this preclinical window, principally amyloid PET [7] and CSF biomarkers [8], are costly, invasive, and poorly suited to population-scale monitoring [9,10]. There is therefore a recognised need for accessible, low-cost, repeatable biomarkers that can support risk stratification and the quantification of disease progression.

The retina is an extension of the central nervous system and offers an optically accessible substrate for studying neurodegeneration in vivo [11]. A growing body of literature reports retinal correlates of AD, including amyloid deposition, vascular changes, and inner retinal thinning, prompting the proposal that the retina can serve as a surrogate for cerebral pathology [12,13,14]. Among retinal compartments, retinal ganglion cells (RGCs) are of particular interest because their dendritic arbours, which stratify within the inner plexiform layer (IPL), appear to be affected early in neurodegeneration [15,16,17]. In the DBA/2J glaucoma model, RGC dendritic atrophy precedes axonal loss and somatic death [18], and in amyloid-overexpressing and knock-in mouse models, including the triple-transgenic AD mouse (3xTg-AD) line used here, RGC dendritic degeneration accompanies synaptic dysfunction and amyloid deposition [15]. The IPL, where these dendrites and their synapses reside, is therefore a biologically relevant region of interest for early detection.

Optical coherence tomography (OCT) is the established modality for non-invasive, micron-scale, cross-sectional retinal imaging and is ubiquitous in ophthalmic practice [19]. Clinically, however, OCT is used almost exclusively to measure layer thickness. Recognising the limitations of thickness-only metrics, recent clinical approaches have begun to incorporate retinal optical texture analysis (ROTA), which integrates thickness and reflectance to visualise glaucomatous nerve fibre bundle defects that are otherwise missed on standard scans [20]. However, even before macroscopic layer dimensions or bundle defects change, early sub-resolution changes in cell and organelle organisation can alter how tissue scatters light long before any measurable change in layer dimensions [21]. The OCT speckle pattern, often discarded as noise, encodes information about the size, density, and refractive index contrast of sub-resolution scatterers such as mitochondria, nuclei, and other organelles [22,23,24]. Quantitative texture analysis [25,26] attempts to recover this information. Grey-level co-occurrence matrix (GLCM) features, which describe the spatial statistics of image intensity, have been used to classify normal from pathological tissue in microscopy, ultrasound, and OCT [27,28], and ultra-high-resolution OCT texture has been shown to track early apoptosis in cultured RGCs, with mitochondrial reorganisation being a likely scattering source [29]. Because OCT speckle arises from the interference of light backscattered by sub-resolution structures, alterations in the size, density, and refractive index contrast of intracellular organelles, such as mitochondria and nuclei, can shift the speckle texture long before any macroscopic change in layer thickness occurs [21,22,23,24].

Methods that detect apoptosing retinal cells using fluorescently labelled annexin V have shown clinical promise but require exogenous contrast agents [30]. A non-invasive image-derived biomarker, extracted from a routine OCT scan, would avoid agent administration, toxicity, and regulatory burden and could be applied retrospectively to existing scans. Whether the subtle, AD-related dendritic changes in the IPL produce a texture signature detectable by OCT and separable by machine learning has not, to our knowledge, been demonstrated.

Here, we address this question in a controlled ex vivo setting. This experimental design is deliberate because the genotype of each explant is known; it provides ground truth for the method, isolating the question of whether an AD-related texture signal exists from the confounds of in vivo acquisition. We imaged retinal explants from 3xTg-AD and C57BL/6 mice with a custom 1040 nm spectral-domain OCT system, extracted GLCM features from IPL volumes of interest (VOIs), and classified them using a support vector machine (SVM) with and without principal component analysis (PCA). We frame this as an exploratory, proof-of-concept study with the purpose of establishing feasibility and defining the design of an in vivo study, rather than estimating diagnostic performance.

## 2. Materials and Methods

**Figure 1:**
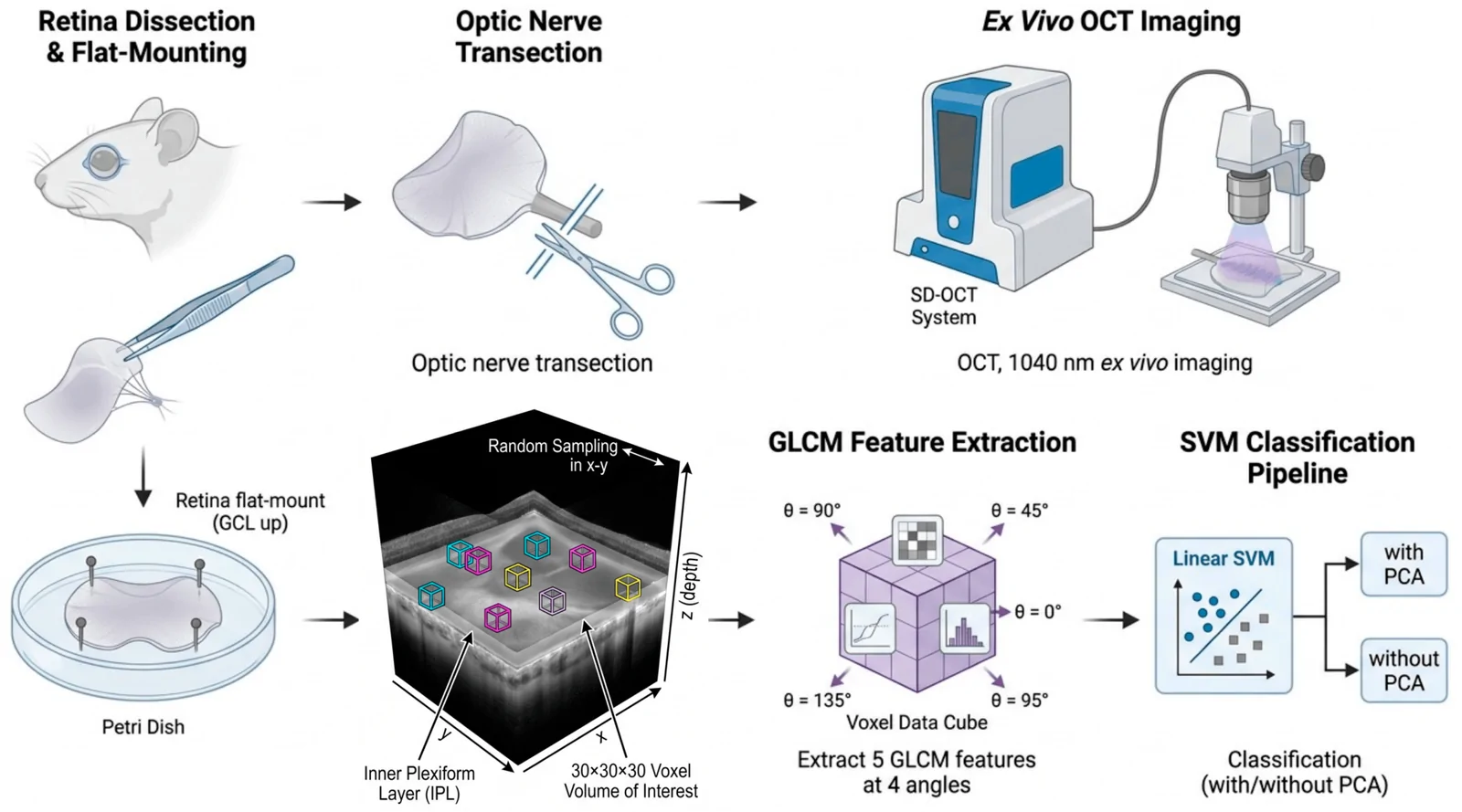
The experimental and analytical pipeline. Flat-mounted retinal explants were imaged with the OCT–microscopy (λ = 1040 nm; FWHM = 70 nm) system immediately after the dissection. The IPL volumes of interest (30 × 30 × 30 voxels) were sampled at random *x*–*y* locations; five textural features at four angles (giving 20 features in total) were extracted and imported to the SVM model for classification purposes (50% of data for training, 25% for validation and 25% for testing), with and without PCA. Abbrev.: OCT—optical coherence tomography; FWHM—full width at half maximum; GCL—ganglion cell layer; IPL—inner plexiform layer; GLCM—grey-level co-occurrence matrix; SVM—support vector machine; PCA—principal component analysis.

All procedures complied with UK Home Office regulations under the Animals (Scientific Procedures) Act 1986 and adhered to the ARVO Statement for the Use of Animals in Ophthalmic and Vision Research. Seven 3xTg-AD mice (The Jackson Laboratory, strain 34830-JAX: B6;129-Tg (APPSwe, tauP301L) 1Lfa Psen1tm1Mpm/Mmjax), aged 12 months, and three C57BL/6J control mice (in-house colony), aged 15 months, were used. Animals were sacrificed by cervical dislocation (Schedule 1). It must be emphasised that the control and AD groups differed in age (15 vs. 12 months), source colony, and background strain (C57BL/6J vs. the mixed B6;129 background of the 3xTg-AD line); therefore, the detected textural differences cannot be attributed solely to AD pathology. Furthermore, due to logistical (animal supply) constraints, the 3xTg-AD and control explants were imaged sequentially in separate groups rather than in a randomised, interleaved order. Because OCT speckle is sensitive to temporal system drift, this non-randomised acquisition order represents a potential confounding batch effect. A schematic of the experimental and analytical pipeline is depicted in Figure 1.

Immediately after sacrifice by cervical dislocation, eyes were enucleated and placed in Hanks’ balanced salt solution (HBSS, Thermo Fisher Scientific Inc., Waltham, MA, USA). The limbus was punctured to remove the anterior segment and lens, and the retina was dissected free and transferred to a Petri dish to be flattened with four radial relieving cuts. Flat-mounted retinae were oriented ganglion cell layer up for imaging. Explants were maintained in HBSS throughout; the imaging of all explants was initiated within 15 min of sacrifice and completed within 45 min to minimise post-mortem tissue degradation; these time windows were consistent across both groups.

### 2.1. OCT Imaging and Processing

A custom research-based spectral-domain OCT–microscopy system was used. The source was an amplified spontaneous emission module (1-M-ASE-HPE-S; NP Photonics, Tucson, AZ, USA) with a central wavelength of λ = 1040 nm and a spectral bandwidth of ~70 nm (full width at half maximum), giving an axial resolution of ~7 µm and a lateral resolution of ~10 µm in air. Light was delivered through a 2 × 2 fibre coupler; the sample arm used a 10× telecentric scan lens (LSM02-BB; Thorlabs, Newton, NJ, USA), providing a >4.7 mm field of view. An attached microscope module aided sample positioning. A total of 20 OCT volumes were acquired across the ten explants (=200 VOIs in total).

**Figure 2:**
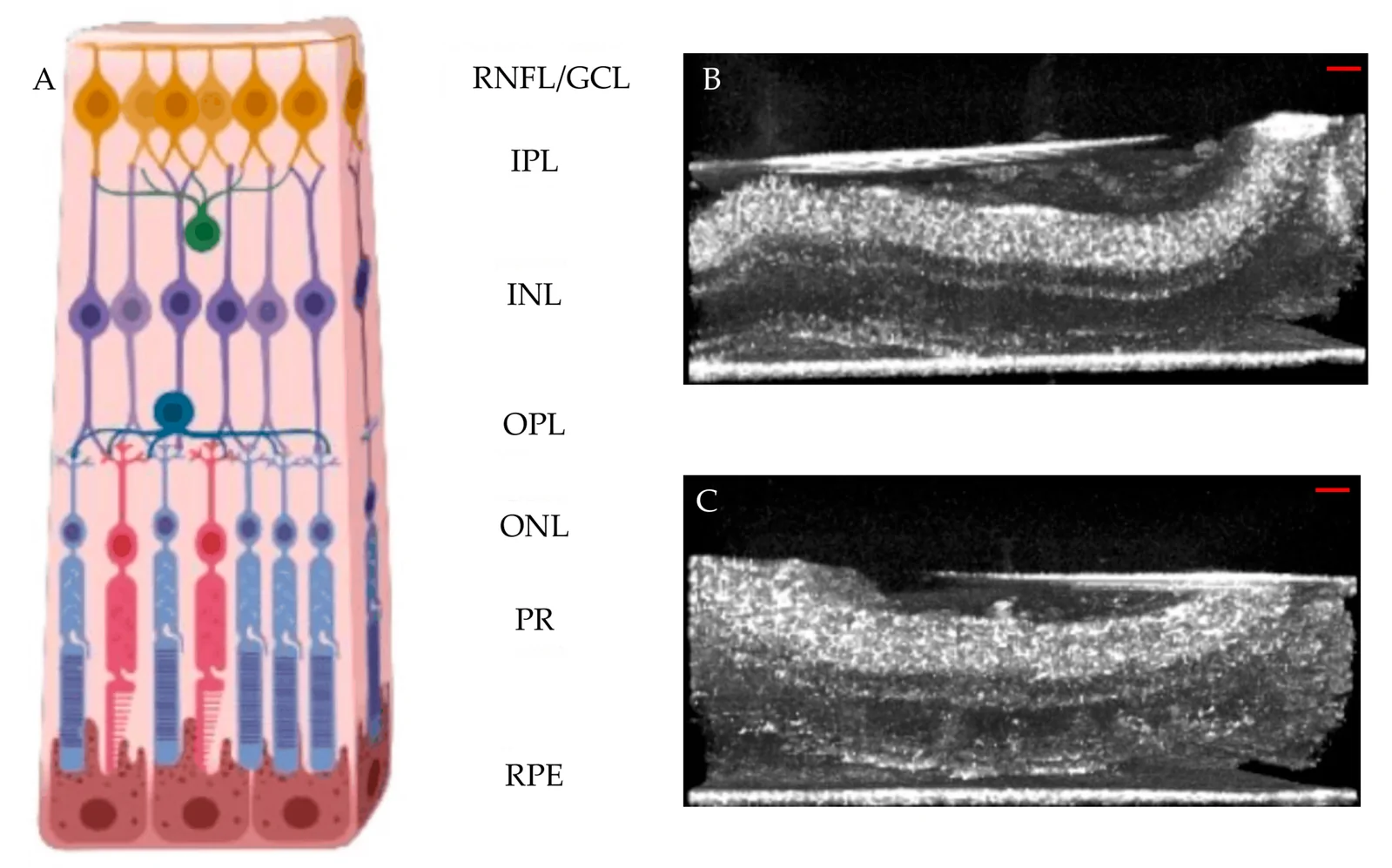
OCT images of flat-mounted retinal explants. (**A**) A schematic of retinal layers corresponding to the OCT cross-sections. (**B**) A representative OCT B-scan from a C57BL/6 control retina. (**C**) A representative OCT B-scan from a 3xTg-AD retina. Scale bars (red, top corner) = 50 μm. The inner plexiform layer (IPL), located between the ganglion cell layer (GCL) and inner nuclear layer (INL), was visually identified for subsequent volumetric sampling. Abbrev.: RNFL—retinal nerve fibre layer; GCL—ganglion cell layer; IPL—inner plexiform layer; INL—inner nuclear layer; OPL—outer plexiform layer; ONL—outer nuclear layer; PR—photoreceptor; RPE—retinal pigment epithelium.

Raw spectral data (FD1 files) were converted to TIFF stacks with dispersion compensation using OCT1_FD1 software (Cardiff University, v2.3). Anomalous B-scans were removed and volumes registered and aligned using an ImageJ macro (v1.51). The IPL was identified from its position between the ganglion cell layer and the inner nuclear layer based on structural morphology (Figure 2A,B). The IPL boundaries were segmented manually by a single experienced operator (M.K.) to ensure intra-observer consistency across all 20 volumes. While gross retinal layer architecture appeared qualitatively comparable between the C57BL/6 and 3xTg-AD explants (Figure 2B,C), sub-resolution textural differences within the IPL formed the basis of the subsequent analysis. Cuboidal VOIs of 30 × 30 × 30 voxels were sampled from the IPL; *x*–*y* placement was randomised using a random number generator (Diplodock, v2.1), and the *z*-extent spanned the IPL. Ten VOIs were sampled per volume, giving 200 VOIs in total across the cohort. The unit of analysis is therefore the VOI (*n* = 200), nested within 20 volumes within 10 animals.

### 2.2. Textural Feature Extraction and Image Classification

Prior to GLCM computation, voxel intensity values within each VOI were linearly normalised to an 8-bit scale (0–255 greyscale levels). Because standard GLCM is inherently two-dimensional, for each 3D VOI, GLCMs were computed on the central orthogonal *x*–*z* and *y*–*z* B-scans and then averaged to represent the 3D volume. Five GLCM features were computed from each VOI: angular second moment (energy/ASM), contrast (inertia), entropy, inverse difference moment (IDM), and correlation [27]. A pixel offset d = 1 was chosen to capture immediate neighbouring voxel dependencies, and four angles (*θ* = 0°, 45°, 90°, 135°) were used to ensure rotational invariance, yielding 20 GLCM properties per VOI. The five features were selected as standard, non-redundant descriptors of texture homogeneity and complexity [27,31]. Texture scripts were adapted from Anantrasirichai et al. (2013) [31] and run in MATLAB R2017b (MathWorks, Natick, MA, USA).

Features were classified using a linear kernel SVM implemented in MATLAB R2017b. A linear kernel was chosen a priori for interpretability and to mitigate overfitting on a small sample, utilising default hyperparameters (BoxConstraint = 1; KernelScale = auto). Two pipelines were compared: SVM on the full 20-feature set and SVM preceded by PCA. Feature selection (identifying contrast and entropy) and PCA fitting were performed exclusively on the training partition to prevent data leakage. The 200 VOIs were partitioned into training (100), validation (50), and test (50) sets. The partition was performed at the VOI level; consequently, VOIs from the same animal could appear across partitions. Therefore, the resulting accuracy estimates the separability of VOIs rather than generalisation to unseen animals.

## 3. Results

Microscopy-based modulation transfer function measurements on retinal explants confirmed that retinal tissue retained optical clarity throughout the full duration of imaging, indicating that texture differences are unlikely to arise from tissue opacification.

**Figure 3:**
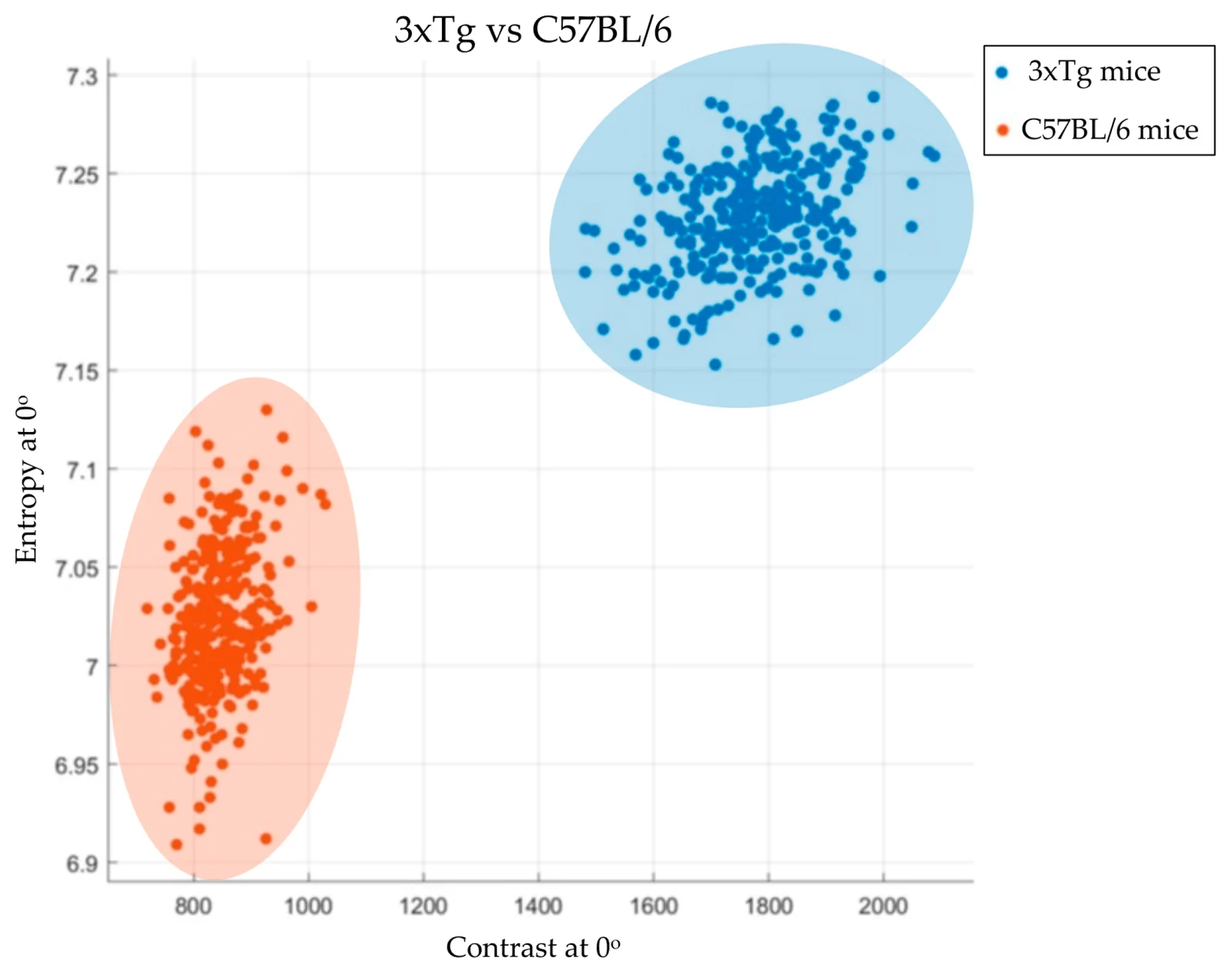
Texture feature space discriminating 3xTg-AD (*n* = 7) from C57BL/6 control (*n* = 3) IPL. In 2D space defined by contrast (0°) and entropy (0°), selected by PCA, all VOIs separate into two non-overlapping clusters. Shaded areas represent 95% confidence ellipses for each group. Linear SVM achieved complete separability with and without PCA.

A texture analysis of IPL VOIs revealed a systematic difference between genotypes. A correlation matrix of all 20 GLCM features revealed high collinearity among angles for the same feature, confirming that discriminative power was concentrated in specific features. Classifiers identified contrast and entropy at 0° as the most descriptive features. In the two-dimensional space defined by these features, the 3xTg-AD and C57BL/6 VOIs formed two completely separable clusters with no overlap (Figure 3). A linear SVM classified OCT images with 100% VOI-level accuracy (sensitivity = 1.00, specificity = 1.00, balanced accuracy = 1.00, ROC-AUC = 1.00; 95% binomial confidence interval for accuracy: 96.4–100%).

Complete VOI-level separability is the central finding: across all 200 sampled VOIs, no misclassifications occurred with either pipeline. The equivalence of the PCA and non-PCA results indicates that the discriminative information is concentrated in a low-dimensional optical signature rather than a diffuse, high-dimensional difference.

## 4. Discussion

This pilot study shows that a quantitative texture analysis of OCT speckle, combined with a simple linear classifier, can discriminate the IPL of 3xTg-AD mice from that of C57BL/6 controls in an ex vivo setting in a two-feature space. The result supports the underlying hypothesis: AD-related changes in the RGC dendritic compartment alter the subtle scattering structure of the IPL in a way that OCT texture can capture, without any exogenous contrast agent. We interpret this as a feasibility demonstration, evidence that a detectable, ligand-free optical signal exists, rather than a prediction of diagnostic performance. Further work is required to correlate changes in optical texture with the quantification of dendritic degeneration within the same tissue sample: an analysis that was outside the scope of this exploratory study.

OCT speckle arises from interference by sub-resolution scatterers whose size, density, and refractive index contrast [26] change as cells degenerate. RGC dendritic atrophy in the IPL involves the loss of synaptic structures and reorganisation of organelles [15,32]; mitochondria, in particular, have been proposed as a dominant scattering source [16,33], and ultra-high-resolution OCT texture has been shown to track staurosporine-induced apoptosis in cultured RGCs, with mitochondrial change being implicated [29]. Nuclear condensation and fragmentation during apoptosis [34] may be further plausible contributors. However, it must be stressed that without paired histology on the same tissue samples, linking these specific textural changes to dendritic pathology remains an inferred hypothesis rather than a direct observation in this dataset. The fact that the discriminative information is concentrated in textural features sensitive to local intensity heterogeneity and disorder is consistent with a shift towards a more heterogeneous, less ordered scattering field, as would be expected from organelle disruption.

SVM-based classification in a pilot study with a small sample size must be carefully considered. First, the unit of analysis should be taken into account: the 200 classified units were VOIs sampled 10 per volume from 20 volumes across 10 animals, and VOIs from the same animal are not independent. Because the train/validation/test partition was made at the VOI level, the same animals contributed to multiple partitions, so the reported accuracy reflects the separability of VOIs and is optimistic with respect to generalisation to unseen animals. A defensible claim is that the classes are separable in this dataset, which signals a strong, reproducible effect, but that animal-level generalisation is not yet established. A leave-one-animal-out analysis [35] would address this directly and is the single most valuable next step to be taken on the existing data.

A further methodological consideration is the sequential, non-interleaved imaging of the two genotype groups. Because OCT speckle and textural features are sensitive to system parameters such as source power and detector sensitivity, any temporal drift in the optical setup during the imaging session could introduce a batch effect. Such an effect could, in principle, contribute to the complete class separation observed here, independent of biological differences. While we have previously demonstrated the optical stability of this specific OCT setup over the duration of typical imaging sessions [16], this non-randomised acquisition order remains a potential confounder in this dataset. Consequently, any future in vivo or confirmatory ex vivo study must employ randomised, interleaved imaging of cases and controls to exclude system drift as a driver of textural differences.

The groups differed not only in genotype but also in age (12 vs. 15 months), source colony, and background strain. Any of these could contribute to a texture difference, so the separation cannot be attributed specifically to AD pathology as opposed to strain, age, or batch effects on retinal microstructure. Although this can be accepted for a feasibility study, it constrains the biological claim and must shape the design of the confirmatory study (age-matched, background-matched controls; multiple cohorts/batches; ideally littermate or genotype control comparisons).

The findings are ex vivo, and whether the signature is detectable in vivo is an open question. A key consideration for clinical translation is the anisotropic resolution of current commercial OCT systems: while the axial resolution is preserved and sufficient to resolve the IPL, the lateral resolution is significantly degraded by ocular aberrations and limited numerical aperture compared to our ex vivo microscopy–OCT setup. While advanced optical designs such as adaptive optics (AO) can improve lateral resolution, they may not be a strict prerequisite for detecting textural changes. For example, Erchova et al. (2018) could detect stimulus-evoked contrast changes in the RGC layer in vivo in tree shrews using through-the-lens imaging with inherently anisotropic resolution [36]. This demonstrates that texture-based analysis can yield viable signals even when lateral resolution is degraded by ocular optics—a principle also exemplified clinically by ROTA [20], which detected glaucomatous defects of the nerve fibre bundle at a macroscopic scale without AO. Nonetheless, the rapid development of next-generation OCT systems using broader-bandwidth light sources [37,38,39] is steadily pushing axial resolution limits further, which may enhance the sensitivity of subcellular [40,41] texture analysis in the future.

A future study could be an in vivo, longitudinal investigation that (1) uses age-, background-, and batch-matched controls; (2) conducts an evaluation with animal-level (leave-one-animal-out) cross-validation and a pre-registered analysis plan; (3) includes paired histological validation; (4) follows AD progression over time to test whether the texture signature tracks disease stage; and (5) extends beyond the IPL to the ganglion cell and nerve fibre layers. Given the small sample size, the non-interleaved acquisition order, and the inter-group differences in age and genetic background, extreme caution is warranted in interpreting these results. This study must be viewed strictly as a methodological demonstration and a hypothesis-generating exercise rather than an estimate of diagnostic performance. Establishing whether the effect survives rigorous controls, and whether it is specific to AD-type pathology versus neurodegeneration generally, is the key open question this pilot raises.

## 5. Conclusions

In an ex vivo proof-of-concept study, textural features extracted from OCT images of the IPL, classified by a linear SVM, demonstrated complete VOI-level separability between 3xTg-AD mice and C57BL/6 controls. Because the method requires no exogenous contrast agent, it is an attractive avenue for future accessible retinal screening. However, extreme caution is warranted: due to the small sample size, VOI-level validation, non-interleaved acquisition, and age/strain mismatches, these results are strictly hypothesis-generating. Definitive follow-up studies requiring in vivo, longitudinal experiments with matched controls, animal-level (leave-one-animal-out) cross-validation, and histological ground truth are mandatory before clinical translation.

## Data Availability

Information on the data underpinning this publication, including access details, can be found in the Figshare Research Data Repository (https://doi.org/10.6084/m9.figshare.32515278).

## Abbreviations

The following abbreviations are used in this manuscript:

3xTg-AD	Triple-transgenic AD mice
AD	Alzheimer’s disease
AO	Adaptive optics
ASM	Angular second moment
FWHM	Full width at half maximum
GCL	Ganglion cell layer
GLCM	Grey-level co-occurrence matrix
HBSS	Hanks’ balanced salt solution
IDM	Inverse difference moment
INL	Inner nuclear layer
IPL	Inner plexiform layer
OCT	Optical coherence tomography
ONL	Outer nuclear layer
OPL	Outer plexiform layer
PCA	Principal component analysis
PET	Positron emission tomography
PR	Photoreceptor
RGC	Retinal ganglion cell
RNFL	Retinal nerve fibre layer
ROTA	Retinal optical texture analysis
RPE	Retinal pigment epithelium
SVM	Support vector machine
